# Symptoms of Posttraumatic Stress Disorder Among Japanese Peacekeepers Deployed in South Sudan

**DOI:** 10.1001/jamanetworkopen.2024.24388

**Published:** 2024-07-24

**Authors:** Masato Kitano, Erik J. Giltay, Taku Saito, Florentine H. S. van der Does, Toshinori Chiba, Eric Vermetten, Naoki Edo, Fumiko Waki, Minori Koga, Hiroyuki Toda, Nic J. van der Wee, Masanori Nagamine

**Affiliations:** 1 Division of Behavioral Science, National Defense Medical College Research Institute, Saitama, Japan; 2 Department of Psychiatry, Leiden University Medical Center, Leiden, the Netherlands; 3 Health Campus The Hague, Leiden University, The Hague, the Netherlands; 4 Department of Psychiatry, Japan Self-Defense Force Hanshin Hospital, Kawanishi, Japan; 5 Department of Psychiatry, National Defense Medical College, Saitama, Japan

## Abstract

**Importance:**

Peacekeepers in United Nations missions experience potentially traumatic events, resulting in increased risk for posttraumatic stress disorder (PTSD). Understanding the course and risk factors of PTSD symptom severity is crucial to ensure personnel safety.

**Objective:**

To investigate the incidence of PTSD, symptom severity trajectories, and potential risk factors associated with adverse trajectories among Japanese peacekeepers deployed in South Sudan.

**Design, Setting, and Participants:**

Data for this 6-year prospective cohort study were collected from December 2011 to December 2018 from Japan Ground Self-Defense Force personnel deployed at the United Nations Mission in South Sudan, corresponding to before and up to 78 months after deployment. Of 3799 potential participants, 80 were excluded because of multiple deployments and 757 because of missing data, incomplete responses, or not providing informed consent. Therefore, 2962 participants were included in the analysis. Data analysis was performed from February 2022 to February 2024.

**Exposure:**

The participants were deployed to United Nations Mission in South Sudan for 6 months.

**Main Outcomes and Measures:**

The 22-item Impact of Event Scale–Revised was used to assess PTSD symptoms, with 25 or more points indicating probable PTSD (p-PTSD). Sociodemographic data were collected, and the 30-item General Health Questionnaire was administered before deployment to identify risk factors for PTSD symptom severity.

**Results:**

In the 2962 participants studied (2901 [97.9%] male; mean [SD] age, 33.9 [7.2] years), the incidence of p-PTSD was 3.95%. Latent growth mixture models identified 4 symptom severity trajectories: resilient (2143 [72.3%]), recovery (479 [16.2%]), protracted (182 [6.1%]), and delayed (158 [5.3%]). Multinomial logistic regression showed that sleep disturbance was a common risk factor for the 2 most severe trajectories (protracted: odds ratio [OR], 1.29; 95% CI, 1.08-1.54; delayed: OR, 1.26; 95% CI, 1.03-1.53), whereas older age (OR, 1.25; 95% CI, 1.06-1.48), anxiety and dysphoria (OR, 1.45; 95% CI, 1.20-1.75), and general illness (OR, 1.30; 95% CI, 1.06-1.59) were associated with the protracted trajectory.

**Conclusions and Relevance:**

This cohort study found that approximately 4% of the participants developed p-PTSD and identified 4 distinct PTSD symptom trajectories. The findings suggest that addressing sleep disturbance and general health issues could effectively prevent PTSD symptoms among peacekeepers.

## Key Points

**Question:**

What are the trajectories and risk factors for posttraumatic stress disorder (PTSD) symptom severity among Japanese peacekeepers deployed to the United Nations Mission in South Sudan?

**Findings:**

This 6-year cohort study of 2962 peacekeepers revealed that 3.95% developed probable PTSD. Multinomial logistic regression revealed that poor predeployment mental health, particularly sleep disturbances, was associated with severe PTSD symptom trajectories, which were identified by latent growth mixture models.

**Meaning:**

These findings suggest that addressing sleep and general health issues before a mission may be effective in preventing PTSD.

## Teaser

This cohort study examines the incidence and risk factors of posttraumatic stress disorder in Japanese peacekeepers engaged in the United Nations Mission in South Sudan.

## Introduction

Peacekeepers deployed to United Nations (UN) peacekeeping operations (PKOs) experience highly stressful situations, including the threat of sudden attacks, the obligation to uphold restraint in dangerous or ambiguous situations, and challenges related to being away from home.^1^ The fatality rate of uniformed peacekeepers has stabilized at less than 1% of those who are deployed.^2^ Nevertheless, 32 peacekeepers were killed in deliberate attacks in 2022.^3^ Peacekeepers are at a higher risk of developing mental disorders, such as depression and posttraumatic stress disorder (PTSD).^4^

Previous studies have reported a PTSD incidence between 1%^5^ and 25.8%^6^ among peacekeepers. A meta-analysis of 12 previous studies on postmission PTSD symptoms in peacekeepers deployed in Haiti, Yugoslavia, South Africa, Lebanon, and Somalia found that the pooled prevalence of PTSD was 5.3% (95% CI, 3.4%-7.2%).^7^ In almost 25% of cases, PTSD symptoms may not develop immediately and may appear months or years after exposure to a traumatic event.^8^

In 1997, the UN established a compensation system for fatalities and permanent impairments. Since 2017, this system has processed more than 400 claims related to PTSD.^9^ Evidently, there is a strong need to prevent PTSD in peacekeepers so that the UN can safely sustain PKOs. Despite PTSD being a major challenge to the safe continuation of the UN PKOs, longitudinal research is lacking,^10^ with only limited and short-term longitudinal studies.

Previous studies have measured the predeployment risk factors for PTSD symptoms specific to the UN PKOs. Brazilian peacekeepers deployed in Haiti reported that their predeployment negative affect was associated with PTSD.^11^ In addition, predeployment psychopathology and negativism have been identified as risk factors among Dutch peacekeepers deployed in Yugoslavia.^12^ Regarding other deployment missions, a meta-analysis revealed sociodemographic status and experiences of traumatic events as predeployment risk factors for PTSD.^13^

The republic of South Sudan became independent from Sudan on July 9, 2011, and the UN worked with the people of South Sudan to protect civilians and build sustained peace. Between January 2012 and May 2017, the Japan Ground Self-Defense Force (JGSDF) dispatched 12 units, totaling 3912 personnel, to participate in the United Nations Mission in South Sudan (UNMISS). Each unit was deployed for 6 months, particularly as a part of the engineering unit. Their main tasks involved improving infrastructure, including that of the UN sites; conducting road repairs; and building facilities for international organizations.^14^ During the JGSDF deployment period, the situation in South Sudan was tense, with friction between the president and former vice president leading to a civil war in 2013^15^ and the escalation of civil war in 2016, resulting in numerous civilian deaths.^16^

Violence against peacekeepers and the resulting fatalities pose challenges for continuing PKOs. Given the importance of the mental health of peacekeepers in the success of PKOs, rigorous long-term follow-up studies are urgently needed. This study aimed to investigate the incidence of PTSD symptoms above the threshold and risk factors associated with severe PTSD symptoms in JGSDF peacekeepers engaged in the UNMISS.

## Methods

### Study Design

This 6-year cohort study was conducted in accordance with the Declaration of Helsinki, and the Strengthening the Reporting of Observational Studies in Epidemiology (STROBE) reporting guideline was followed. It was approved by the Ethics Committee of the National Defense Medical College. Written informed consent was obtained, or the opportunity to opt out was provided.

The study was conducted in JGSDF personnel deployed at the UNMISS. General health data were collected during the training period, approximately 1 month before deployment, starting from Decemer 2011. The deployment candidates were screened through physical and mental checks. Posttraumatic stress disorder symptoms were measured as a part of the annual Ministry of Defense Employee Mental Health Assessment conducted from October 2013 to December 2018. We matched the predeployment general health data with annual PTSD symptom data on an individual basis. Because the units were deployed at different time points, we aligned the mission completion and return to Japan to time zero. This approach allowed us to convert the time of data collection to the elapsed time since the end of deployment (eFigure 1 in Supplement 1).

Civilian members and those who were absent during the data collection period were excluded. The initial pool of eligible participants consisted of 3799 JGSDF members. Only first-deployment data were included for participants who had been deployed multiple times, which excluded 80 responses. In addition, 757 participants were excluded for not responding to or answering the Impact of Event Scale–Revised (IES-R) fewer than 2 times, not providing informed consent, or having missing values. Finally, data on 2962 participants were included in the analysis (eFigure 2 in Supplement 1).

### Measures

The demographic information included age, sex, and rank. General psychological health was assessed using the 30-item General Health Questionnaire (GHQ-30), a self-administered questionnaire on minor psychiatric disorders. Responses were rated on a 4-point Likert scale (with 1 indicating not at all and 4 indicating much more than usual), with a total score ranging from 30 to 120.^17,18^ The reliability and validity of the GHQ-30 Japanese version have been confirmed in the Japanese population.^19,20^ In the current study, the overall internal consistency was high (Cronbach α = 0.91). The general illness subscale (McDonald ω = 0.72) indicated the tendency of pathology, the somatic symptom subscale (McDonald ω = 0.75) indicated physical disturbance, the sleep disturbance subscale (McDonald ω = 0.72) indicated insomnia and nightmare, the social dysfunction subscale (McDonald ω = 0.68) indicated the degree of activity, the anxiety and dysphoria subscale (McDonald ω = 0.86) indicated the tendency of anxiety and dysphoria, and the suicidal depression subscale (McDonald ω = 0.90) indicated the tendency of suicidal attempt and depression. Each subscale demonstrated sufficient internal consistency, making it a reliable measurement tool.

Severity of PTSD symptoms was assessed using the Japanese version of the IES-R,^21,22^ which demonstrated high internal consistency (Cronbach α = 0.93) in the current cohort. The IES-R consists of 22 items (eg, “any reminder brings back feelings about it” and “I have trouble staying asleep”). Responses were scored on a 5-point Likert scale (with 0 indicating not at all and 4 indicating extremely), with the total score ranging from 0 to 88.^23^ This questionnaire was administered up to 6 times per participant at the annual mental health assessment and was converted into an elapsed time scale of 12 points. An IES-R score of 25 or higher indicated a probable PTSD (p-PTSD) diagnosis.^21^ A previous study also found good psychometric properties of the IES-R.^24^

### Statistical Analysis

The demographic characteristics of the participants were compared by conducting a χ^2^ test and a 1-way analysis of variance test in each trajectory group. We confirmed the data completeness by summarizing each time point and using a correlation coefficient matrix (eTables 1 and 2 in Supplement 1). We counted the cumulative number of p-PTSD incidents during the follow-up period. To address the positively skewed distribution of the IES-R total scores, the data were normalized using a natural logarithmic transformation before analysis. We used latent growth mixture models (LGMM) to identify the PTSD symptom severity trajectory groups by classifying the trajectories of the log_e_-transformed IES-R total score with a linear mixture model with 100 iterations from 500 random departures by the gridsearch function. We modeled the analysis using 1 to 5 clusters and determined the appropriate model using the Akaike information criterion (AIC), the bayesian information criterion (BIC), and sample size–adjusted BIC (SBIC). Entropy was used to assess the discriminatory power of the models. The best fit was determined by the lowest AIC, BIC, and SBIC, with entropy values approaching 1.^25^ The initial value from the maximum likelihood estimates of a 1-class model was used for the multiclass models. Subsequently, we analyzed the factors associated with each PTSD symptom severity trajectory using multivariate multinomial logistic regression (MLR), with the resilient trajectory as the reference group. The independent variables were standardized before analyses to directly compare the strength of the odds ratios (ORs) with 95% CIs.

All statistical analyses were performed using R, version 4.2.2 and RStudio, version 2022.12.0.353 (R Foundation).^26,27^ The main packages used were lcmm, version 1.9.5 for LGMM^28^ and nnet, version 7.3.18 for MLR.^29^ The threshold for statistical significance was set at a 2-sided *P* < .05. Data analysis was performed from February 2022 to February 2024.

## Results

### Participants

Of the 2962 participants (2901 [97.9%] male and 61 [2.1%] female; mean [SD] age, 33.9 [7.2 years]), 536 (18.1%) were officers, 2205 (74.4%) were sergeants, and 221 (7.5%) were privates. The demographic characteristics of the participants are summarized in the Table.

**Table.** Predeployment Sociodemographic Variables and General Health Questionnaire (GHQ) Scores for Each Trajectory Group^a^

Variable	Total (N = 2962)	Resilient (n = 2143)	Recovery (n = 479)	Protracted (n = 182)	Delayed (n = 158)	*P* value
Age, mean (SD), y	33.9 (7.2)	33.3 (7.1)	35.3 (7.4)	36.2 (7.3)	34.2 (6.6)	<.001
Sex, No. (%)						
Male	2901 (97.9)	2109 (98.4)	462 (96.5)	177 (97.3)	153 (96.8)	.03
Female	61 (2.1)	34 (1.6)	17 (3.5)	5 (2.7)	5 (3.2)	
Rank, No. (%)						
Sergeant	2205 (74.4)	1626 (75.9)	336 (70.1)	125 (68.7)	118 (74.7)	<.001
Private	221 (7.5)	184 (8.6)	23 (4.8)	6 (3.3)	8 (5.1)	
Officer	536 (18.1)	333 (15.5)	120 (25.1)	51 (28.0)	32 (20.3)	
GHQ scores, mean (SD)						
General illness	8.2 (2.0)	7.9 (1.9)	8.6 (2.1)	9.3 (2.4)	8.6 (2.2)	<.001
Somatic symptom	6.5 (1.9)	6.3 (1.7)	7 (2.1)	7.4 (2.3)	6.8 (2.1)	<.001
Sleep disturbance	7.7 (2.5)	7.4 (2.3)	8.3 (2.6)	9.2 (3.0)	8.3 (2.7)	<.001
Social dysfunction	9.4 (1.6)	9.4 (1.6)	9.6 (1.6)	9.7 (1.8)	9.5 (1.7)	.005
Anxiety and dysphoria	7.2 (2.3)	6.9 (2.2)	7.8 (2.4)	8.7 (2.7)	7.6 (2.2)	<.001
Suicidal depression	5.5 (1.3)	5.4 (1.1)	5.7 (1.5)	6.2 (2.1)	5.7 (1.7)	<.001

^a^ The *P* value was calculated by conducting a χ^2^ test for categorical variables (sex and rank) and 1-way analysis of variance test for continuous variables (age and subscale scores of the GHQ), whereas all were compared among the 4 groups, except for the total column.

### Main Outcomes

**Figure 1.**
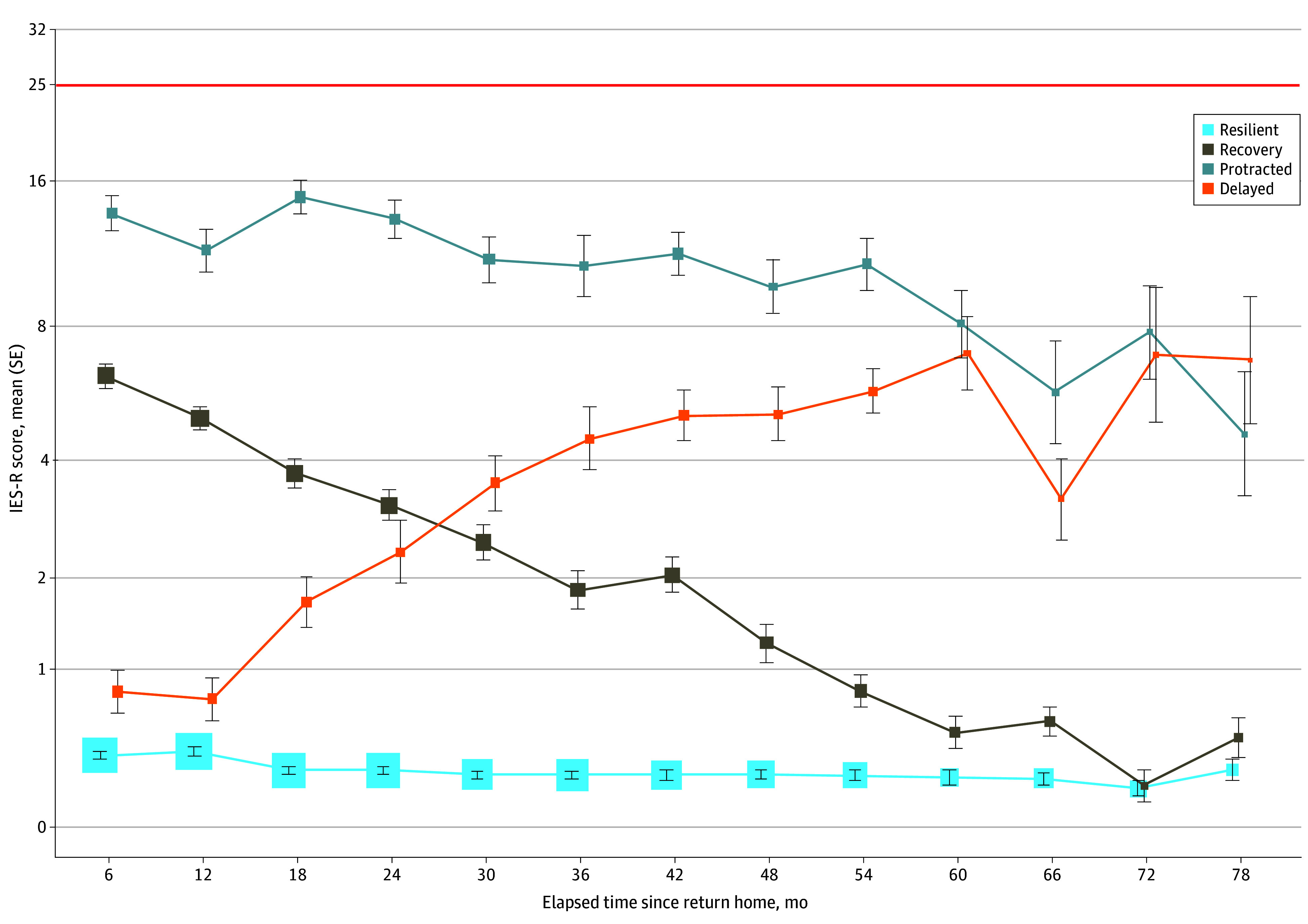
Longitudinal Trajectories for Impact of Event Scale–Revised (IES-R) by Latent Growth Mixture Models The size of the squares is proportional to the number of participants whose data are in the category at each time point. Error bars indicate SEs; red line, the cutoff score of probable posttraumatic stress disorder.

The cumulative incidence of p-PTSD was 3.95% (n = 117) in the 78 months of follow-up. On the basis of the LGMM results, a 4-trajectory model was selected as the best fit, with the smallest AIC (22624.35), BIC (22696.27), SBIC (22658.14), and entropy (0.82) (eFigure 3 in Supplement 1). The trajectories were named resilient (n = 2143 [72.3%]), recovery (n = 479 [16.2%]), protracted (n = 182 [6.1%]), and delayed (n = 158 [5.3%]) based on the longitudinal outcomes. The resilient group had fewer PTSD symptoms throughout the study period. The recovery group initially displayed some PTSD symptoms after deployment, which gradually decreased and reached levels similar to those of the resilient group at 78 months. The protracted group initially had higher levels of PTSD symptoms than the other trajectory groups; the PTSD symptoms decreased but did not reach the same level as that of the recovery group at the end of the 6-year follow-up. The delayed group initially had low levels of PTSD symptoms, which gradually increased (Figure 1).

**Figure 2.**
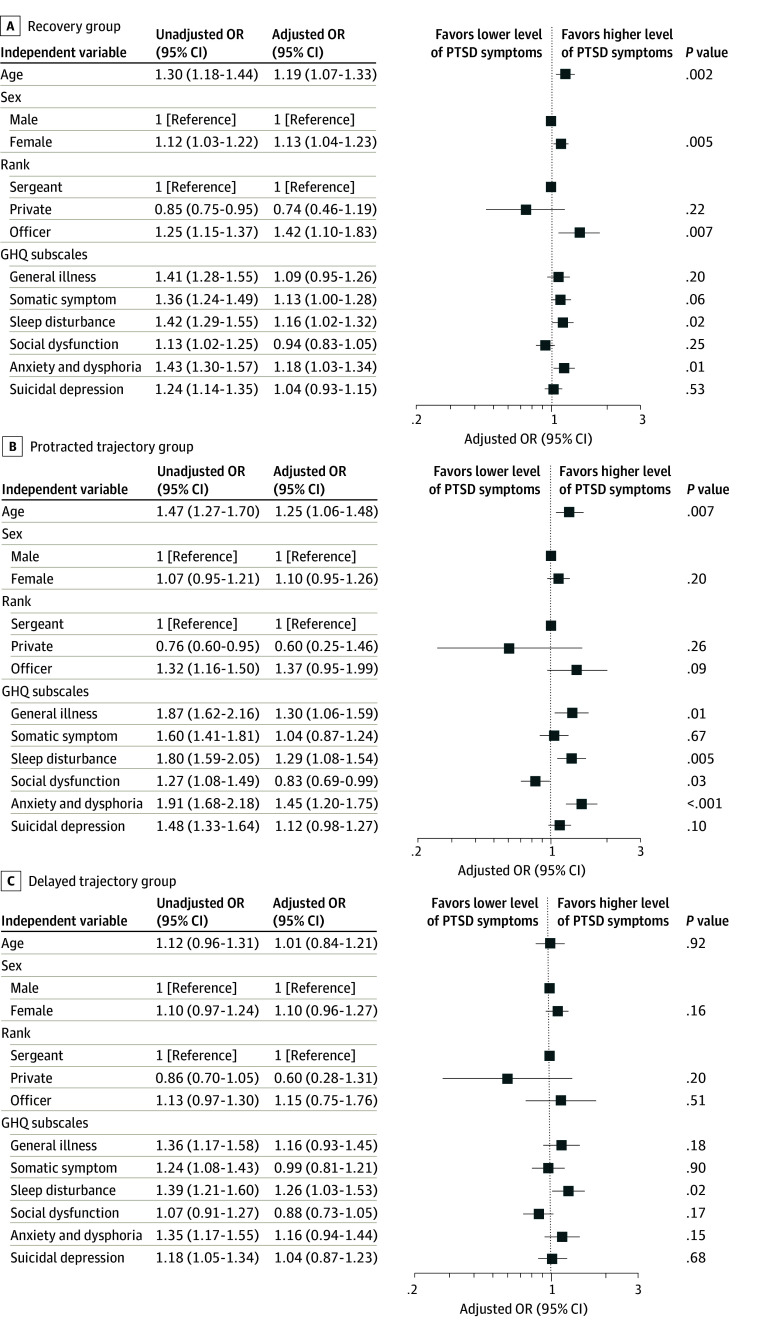
Odds Ratios (ORs) for the Recovery, Protracted, and Delayed Trajectory Groups by Multinomial Logistic Regression Error bars indicate 95% CIs. GHQ indicates General Health Questionnaire.

Subsequently, we conducted MLR using the resilient group as a reference to examine the factors associated with each group. For the recovery group, older age (OR, 1.19; 95% CI, 1.07-1.33; *P* = .002), female sex (OR, 1.13; 95% CI, 1.04-1.23; *P* = .005), officer rank (OR, 1.42; 95% CI, 1.10-1.83; *P* = .007), and predeployment sleep disturbance (OR, 1.16; 95% CI, 1.02-1.32; *P* = .02) and anxiety and dysphoria (OR, 1.18; 95% CI, 1.03-1.34; *P* = .01) on the GHQ-30 were significantly associated with the symptomatic trajectories (Figure 2A). For the protracted group, older age (OR, 1.25; 95% CI, 1.06-1.48; *P* = .007) and predeployment general illness (OR, 1.30; 95% CI, 1.06-1.59; *P* = .01), sleep disturbance (OR, 1.29; 95% CI, 1.08-1.54; *P* = .005), social dysfunction (OR, 0.83; 95% CI, 0.69-0.99; *P* = .03), and anxiety and dysphoria (OR, 1.45; 95% CI, 1.20-1.75; *P* < .001) were significantly associated factors (Figure 2B). For the delayed group, sleep disturbance (OR, 1.26; 95% CI, 1.03-1.53; *P* = .02) on the predeployment GHQ-30 was a significantly associated factor (Figure 2C). The variance inflation factors for each independent variable in the current multinomial model ranged from 1.13 to 2.10.

## Discussion

This study aimed to examine the incidence of PTSD in JGSDF personnel deployed to the UNMISS and identify symptom severity trajectories and their risk factors. The 6-year cumulative p-PTSD incidence was approximately 4%, and 4 distinct trajectories were identified: resilient, recovery, protracted, and delayed. The most significant predeployment risk factors for PTSD symptom severity were sleep disturbances and poor mental health.

The approximately 4% incidence of p-PTSD in the current study was lower than the 5.3% PTSD prevalence reported in a previous meta-analysis^7^ of 12 studies on peacekeepers. On the basis of Japan’s Constitution, which stipulates renunciations of war, the activities of the JGSDF in PKO are limited to cease-fire zones, prohibiting participation in combat operations. Owing to these restrictions, JGSDF peacekeepers may have experienced less combat exposure than peacekeepers in other countries. However, the political situation in South Sudan was unstable during the period of JGSDF operations,^15,16^ with many stressors, such as the spread of civil war and the threat of hostile attacks by local people. In addition, the current study’s method of a long-term follow-up survey may have enhanced the detection of p-PTSD.

To the best of our knowledge, this is the first report of a trajectory analysis of PTSD symptoms in UN peacekeepers. Although most of the JGSDF personnel in the UNMISS experienced few or transient PTSD symptom severity trajectories (resilient: 72.3%, recovery: 16.2%), more than 10% (protracted: 6.1%, delayed: 5.3%) presented with more severe symptom trajectories. These findings highlight the importance of providing special attention and support to the 2 subgroups (protracted and delayed) of peacekeepers because they may be at a higher risk of experiencing long-term psychological challenges. The results were consistent with the findings of a review^30^ that analyzed 54 research articles on PTSD symptom trajectory. It identified 4 prototype symptom trajectories that could result from potentially traumatic events: resilience (mean of 65.7% across populations), recovery (20.8%), chronicity (10.6%), and delayed onset (8.9%). The prevalence of each trajectory in this study was similar to these values. Similar prevalence and trajectories were also found in a previous study^31^ on JGSDF first responders deployed to the 2011 Great East Japan Earthquake, although the traumatic events were different.

In this study, predeployment sleep disturbance was identified as a common risk factor for protracted and delayed groups, which is consistent with the findings of previous studies.^32,33,34^ A previous study of Dutch veterans who had been deployed in Afghanistan showed that predeployment nightmares were a risk factor for developing PTSD after deployment.^35^ Sleep disturbances before deployment may increase susceptibility to traumatic stress during deployment because it may compromise the ability to effectively handle stressful situations. It has also been suggested that PTSD and insomnia symptoms have a bidirectional association and may be mutually maintained or exacerbated.^36,37^ Sleep disturbances can impair the normal processing and consolidation of memories and emotions, making it difficult to effectively cope with traumatic experiences. The present findings implied that the sleep condition before deployment might have long-term effects on individuals’ lives.

General illness and anxiety and dysphoria on the GHQ-30 before deployment were identified as associated factors in the protracted group. Low mental and physical health status before deployment to a war zone was identified as a risk factor for PTSD in the Millennium Cohort Study in the United States,^38,39^ and pretrauma psychopathology has been described as a risk factor for PTSD.^40^ In a machine learning–based study, depressive and anxiety symptoms were identified as important factors associated with symptomatic PTSD severity trajectories with high sensitivity and specificity.^41^ These findings, including the results of this study, highlight the importance of conducting general health screenings for UN peacekeepers before deployment. Low mental or physical health status may worsen a vulnerable psychological state, making individuals more susceptible to traumatic stress during their mission and increasing the risk of developing PTSD symptoms. Proactive educational or medical interventions for those at risk for PTSD, identified during the predeployment screening, could have a positive effect on the prevention or alleviation of PTSD symptoms among peacekeepers.

Older individuals were more likely to be in the protracted trajectory group. This finding is inconsistent with the findings of a study on Dutch UN peacekeepers who participated in the UN Protection Force mission in the former Yugoslavia, which reported that younger age was significantly associated with PTSD symptom severity.^12^ A meta-analysis on the risk factors for combat-related PTSD did not show age to be a significant factor,^13^ which implies that the findings might vary depending on the study cohorts. Our findings may be an artifact of the JGSDF employment system, in which employment is permanent. Older members of the JGSDF generally have a long service history and tend to have more experience, not only as first responders in Japan, where disasters occur frequently, but also as UN peacekeepers; therefore, they are more likely to be exposed to potentially traumatic events. This may have acted as a potential confounding factor,^13^ with older age serving as a proxy for cumulative traumatic experiences. A previous study also found that older age was a factor associated with postdeployment p-PTSD^42^ and more adverse PTSD symptom severity trajectories in JGSDF personnel deployed after the Great East Japan Earthquake, possibly reflecting this organizational structure.^31^

### Limitations

The present study has some limitations. First, although the participants underwent a general medical check before deployment, they were not screened for PTSD symptoms, raising the possibility that some may have had p-PTSD before deployment. Second, the data were collected as a part of organizational mental health management. Therefore, data that could have provided more insight into the population were not collected, and known risk factors for the development of PTSD were not assessed. These data included potential traumatic events (before, during, and after deployment), medical history, alcohol and substance abuse, and social support after the mission. Third, because the data were collected as an occupational health survey rather than as an anonymous survey, participants may have underreported their symptoms.^43^ Fourth, significant attrition was observed throughout the study. Participants with severe psychological conditions may have been retired or unable to respond to the health survey, potentially leading to an underestimation of the incidence of p-PTSD (eTable 3 in Supplement 1). Fifth, because this survey used only a self-report questionnaire, it could not reveal the exact PTSD diagnosis. Sixth, the number of female JGSDF personnel deployed at the UNMISS was low, limiting the generalizability of our findings to female peacekeepers.

## Conclusions

In this longitudinal study of the JGSDF personnel deployed at the UNMISS, we examined the cumulative incidence of p-PTSD and identified 4 trajectories of PTSD symptom severity. Subsequently, we identified the following predeployment general health factors associated with symptomatic trajectories: sleep disturbance, anxiety and dysphoria, and general illness. These predeployment health issues may influence the later development of clinical PTSD symptoms. Peacekeepers’ mental health is essential for sustained and effective implementation of the UN PKOs. These findings provide valuable information on how PTSD symptoms may develop after peacekeeping missions and who may be at risk. Moreover, given these findings, UN member-state policymakers should consider developing predeployment mental health screenings and educational or medical interventions aimed at preventing PTSD symptoms among peacekeepers.

## Notes

Supplement 1. **eTable 1.** Summary and Transition Table of the Impact of Event Scale-Revised (IES-R) During the Study Time Frame **eTable 2.** Pearson Correlation Coefficient Matrix Within the Subscale of the General Health Questionnaire (GHQ) **eTable 3.** Comparison of Variables Between Participants Who Provided Data Once and Those Who Provided Data Twice or More **eFigure 1.** Time Transformation in the Dataset With Deployment and Follow-Up-Collected Time-to-Event Times **eFigure 2.** Flowchart of the Selection of Participants for the Analysis **eFigure 3.** Fit Indices of the Impact of Event Scale-Revised (IES-R) Assessments in Latent Growth Mixture Models (LGMM)

Supplement 2.
Data Sharing Statement

## References

[1] United Nations Department of Peacekeeping Operations. *United Nations Stress Management Booklet*. United Nations Department of Peace-Keeping Operations; 1995. Accessed June 8, 2024. https://www.navedu.navy.mi.th/stg/databasestory/data/laukniyom/workjob/bigcountry-workjob/UN-Publications/082-un_stress_management_booklet.pdf

[2] Van Der Lijn J, Smit T. Peacekeepers under threat? fatality trends in un peace operations. Stockholm International Peace Research Institute (SIPRI) Policy Brief. Accessed June 8, 2024. https://www.sipri.org/publications/2015/sipri-fact-sheets/peacekeepers-under-threat-fatality-trends-un-peace-operations

[3] United Nations. At Least 32 Peacekeeping, Associated Personnel Killed in Malicious Attacks During 2022, United Nationals Staff Union President Says. Accessed September 26, 2023. https://press.un.org/en/2023/org1730.doc.htm

[4] Forbes D, O’Donnell M, Brand RM, . The long-term mental health impact of peacekeeping: prevalence and predictors of psychiatric disorder. BJPsych Open. 2016;2(1):32-37. doi:10.1192/bjpo.bp.115.001321 27703751 PMC4995565

[5] Macdonald C, Chamberlain K, Long N, Mirfin K. Stress and mental health status associated with peacekeeping duty for New Zealand Defence Force personnel. Stress Med. 1999;15(4):235-241. doi:10.1002/(SICI)1099-1700(199910)15:4<235::AID-SMI819>3.0.CO;2-U

[6] Seedat S, le Roux C, Stein DJ. Prevalence and characteristics of trauma and post-traumatic stress symptoms in operational members of the South African National Defence Force. Mil Med. 2003;168(1):71-75. doi:10.1093/milmed/168.1.71 12546250

[7] Souza WF, Figueira I, Mendlowicz MV, . Posttraumatic stress disorder in peacekeepers: a meta-analysis. J Nerv Ment Dis. 2011;199(5):309-312. doi:10.1097/NMD.0b013e3182175180 21543949

[8] Smid GE, Mooren TTM, van der Mast RC, Gersons BPR, Kleber RJ. Delayed posttraumatic stress disorder: systematic review, meta-analysis, and meta-regression analysis of prospective studies. J Clin Psychiatry. 2009;70(11):1572-1582. doi:10.4088/JCP.08r04484 19607763

[9] Uniformed Capabilities Support Division. Comprehensive Study to Develop a PTSD Framework for Uniformed Personnel Final Study Report. Dept of Operational Support; 2021.

[10] Gray MJ, Bolton EE, Litz BT. A longitudinal analysis of PTSD symptom course: delayed-onset PTSD in Somalia peacekeepers. J Consult Clin Psychol. 2004;72(5):909-913. doi:10.1037/0022-006X.72.5.909 15482050

[11] Souza WF, Figueira I, Mendlowicz MV, . Negative affect predicts posttraumatic stress symptoms in Brazilian volunteer United Nations peacekeepers in Haiti. J Nerv Ment Dis. 2008;196(11):852-855. doi:10.1097/NMD.0b013e31818b4682 19008738

[12] Bramsen I, Dirkzwager AJ, van der Ploeg HM. Predeployment personality traits and exposure to trauma as predictors of posttraumatic stress symptoms: a prospective study of former peacekeepers. Am J Psychiatry. 2000;157(7):1115-1119. doi:10.1176/appi.ajp.157.7.1115 10873920

[13] Xue C, Ge Y, Tang B, . A meta-analysis of risk factors for combat-related PTSD among military personnel and veterans. PLoS One. 2015;10(3):e0120270. doi:10.1371/journal.pone.0120270 25793582 PMC4368749

[14] Ministry of Defense. South Sudan PKO (UNMISS). Accessed September 23, 2023. https://warp.da.ndl.go.jp/info:ndljp/pid/11591426/www.mod.go.jp/e/d_act/kokusai_heiwa/pko/s_sudan_pko/index.html

[15] BBC News. South Sudan opposition head Riek Machar denies coup bid. December 18, 2013. Accessed October 26, 2023. https://www.bbc.co.uk/news/world-africa-25427619.

[16] BBC News. South Sudan “back to war,” says VP Riek Machar’s spokesman. July 10, 2016. Accessed October 26, 2023. https://www.bbc.com/news/world-africa-36758013

[17] Goldberg DP, Rickels K, Downing R, Hesbacher P. A comparison of two psychiatric screening tests. Br J Psychiatry. 1976;129(1):61-67. doi:10.1192/bjp.129.1.61 938806

[18] Goldberg DP, Hillier VF. A scaled version of the General Health Questionnaire. Psychol Med. 1979;9(1):139-145. doi:10.1017/S0033291700021644 424481

[19] Iwata N, Uno B, Suzuki T. Psychometric properties of the 30-item version general health questionnaire in Japanese. Jpn J Psychiatry Neurol. 1994;48(3):547-556. doi:10.1111/j.1440-1819.1994.tb03013.x 7891417

[20] Kitamura T, Sugawara M, Aoki M, Shima S. Validity of the Japanese version of the GHQ among antenatal clinic attendants. Psychol Med. 1989;19(2):507-511. doi:10.1017/S0033291700012538 2762449

[21] Asukai N, Kato H, Kawamura N, . Reliability and validity of the Japanese-language version of the impact of event scale-revised (IES-R-J): four studies of different traumatic events. J Nerv Ment Dis. 2002;190(3):175-182. doi:10.1097/00005053-200203000-00006 11923652

[22] Weiss DS. The Impact of Event scale: revised. In: Tang CS, ed. Cross-Cultural Assessment of Psychological Trauma and PTSD. Springer Science+Business Media; 2007:219-238. doi:10.1007/978-0-387-70990-1_10

[23] Weiss D, Marmar C. The impact of event scale—revised. In: Wilson JP, Keane TM, eds. Assessing Psychological Trauma and PTSD. Guilford Press; 1997:399-411.

[24] Arnberg FK, Michel PO, Johannesson KB. Properties of Swedish posttraumatic stress measures after a disaster. J Anxiety Disord. 2014;28(4):402-409. doi:10.1016/j.janxdis.2014.02.005 24726240

[25] Jung T, Wickrama KAS. An introduction to latent class growth analysis and growth mixture modeling. Soc Personal Psychol Compass. 2008;2(1):302-317. doi:10.1111/j.1751-9004.2007.00054.x

[26] R Core Team. R: A language and environment for statistical computing. R Foundation for Statistical Computing; 2022. Accessed June 8, 2024. https://www.R-project.org/

[27] Posit Team. RStudio: Integrated Development Environment for R. Posit Software; 2022. Accessed June 8, 2024. http://www.posit.co/

[28] Proust-Lima C, Philipps V, Liquet B. Estimation of extended mixed models using latent classes and latent processes: the R package lcmm. arXiv. J Stat Softw. 2015;78(2). doi:10.18637/jss.v078.i02

[29] Ziegel ER. Modern applied statistics with S. Technometrics. 2003;45(1):111. doi:10.1198/tech.2003.s33

[30] Galatzer-Levy IR, Huang SH, Bonanno GA. Trajectories of resilience and dysfunction following potential trauma: a review and statistical evaluation. Clin Psychol Rev. 2018;63:41-55. doi:10.1016/j.cpr.2018.05.008 29902711

[31] Saito T, van der Does FHS, Nagamine M, . Risk and resilience in trajectories of post-traumatic stress symptoms among first responders after the 2011 Great East Japan Earthquake: 7-year prospective cohort study. Br J Psychiatry. 2022:1-8. doi:10.1192/bjp.2022.2 35191369

[32] Wang HE, Campbell-Sills L, Kessler RC, . Pre-deployment insomnia is associated with post-deployment post-traumatic stress disorder and suicidal ideation in US Army soldiers. Sleep. 2019;42(2):zsy229. doi:10.1093/sleep/zsy229 30508139 PMC6369721

[33] Acheson DT, Kwan B, Maihofer AX, . Sleep disturbance at pre-deployment is a significant predictor of post-deployment re-experiencing symptoms. Eur J Psychotraumatol. 2019;10(1):1679964. doi:10.1080/20008198.2019.1679964 31723377 PMC6830277

[34] Koffel E, Polusny MA, Arbisi PA, Erbes CR. Pre-deployment daytime and nighttime sleep complaints as predictors of post-deployment PTSD and depression in National Guard troops. J Anxiety Disord. 2013;27(5):512-519. doi:10.1016/j.janxdis.2013.07.003 23939336

[35] van Liempt S, van Zuiden M, Westenberg H, Super A, Vermetten E. Impact of impaired sleep on the development of PTSD symptoms in combat veterans: a prospective longitudinal cohort study. Depress Anxiety. 2013;30(5):469-474. doi:10.1002/da.22054 23389990

[36] van Liempt S. Sleep disturbances and PTSD: a perpetual circle? Eur J Psychotraumatol. 2012;3(1):19142. doi:10.3402/ejpt.v3i0.19142 23050070 PMC3464455

[37] Kartal D, Arjmand HA, Varker T, . Cross-lagged relationships between insomnia and posttraumatic stress disorder in treatment-receiving veterans. Behav Ther. 2021;52(4):982-994. doi:10.1016/j.beth.2020.12.006 34134836

[38] Sandweiss DA, Slymen DJ, Leardmann CA, ; Millennium Cohort Study Team. Preinjury psychiatric status, injury severity, and postdeployment posttraumatic stress disorder. Arch Gen Psychiatry. 2011;68(5):496-504. doi:10.1001/archgenpsychiatry.2011.44 21536979

[39] LeardMann CA, Smith TC, Smith B, Wells TS, Ryan MAK; Millennium Cohort Study Team. Baseline self reported functional health and vulnerability to post-traumatic stress disorder after combat deployment: prospective US military cohort study. BMJ. 2009;338:b1273. doi:10.1136/bmj.b1273 19372117 PMC2671472

[40] DiGangi JA, Gomez D, Mendoza L, Jason LA, Keys CB, Koenen KC. Pretrauma risk factors for posttraumatic stress disorder: a systematic review of the literature. Clin Psychol Rev. 2013;33(6):728-744. doi:10.1016/j.cpr.2013.05.002 23792469

[41] Schultebraucks K, Qian M, Abu-Amara D, . Pre-deployment risk factors for PTSD in active-duty personnel deployed to Afghanistan: a machine-learning approach for analyzing multivariate predictors. Mol Psychiatry. 2021;26(9):5011-5022. doi:10.1038/s41380-020-0789-2 32488126 PMC8589682

[42] Nagamine M, Giltay EJ, Shigemura J, . Assessment of factors associated with long-term posttraumatic stress symptoms among 56 388 first responders after the 2011 Great East Japan Earthquake. JAMA Netw Open. 2020;3(9):e2018339. doi:10.1001/jamanetworkopen.2020.18339 32990742 PMC7525349

[43] McLay RN, Deal WE, Murphy JA, Center KB, Kolkow TT, Grieger TA. On-the-record screenings versus anonymous surveys in reporting PTSD. Am J Psychiatry. 2008;165(6):775-776. doi:10.1176/appi.ajp.2008.07121960 18519540

