# Supplementary material for: Symptoms of Posttraumatic Stress Disorder Among Japanese Peacekeepers Deployed in South Sudan
Source: JAMA Netw Open. 2024 Jul 24;7(7):e2424388. doi: 10.1001/jamanetworkopen.2024.24388 (PMC11270132; doi:10.1001/jamanetworkopen.2024.24388)
Supplement: Supplement 1. — eTable 1. Summary and Transition Table of the Impact of Event Scale-Revised (IES-R) During the Study Time Frame eTable 2. Pearson Correlation Coefficient Matrix Within the Subscale of the General Health Questionnaire (GHQ) eTable 3. Comparison of Variables Between Participants Who Provided Data Once and Those Who Provided Data Twice or More eFigure 1. Time Transformation in the Dataset With Deployment and Follow-Up-Collected Time-to-Event Times eFigure 2. Flowchart of the Selection of Participants for the Analysis eFigure 3. Fit Indices of the Impact of Event Scale-Revised (IES-R) Assessments in Latent Growth Mixture Models (LGMM) [file jamanetwopen-e2424388-s001.pdf]

Supplementary Online Content

Kitano M, Giltay EJ, Saito T, et al. Symptoms of posttraumatic stress disorder among Japanese peacekeepers deployed in South Sudan. *JAMA Netw Open*. 2024;7(7):e180818. doi:10.1001/jamanetworkopen.2018.0818

eTable 1. Summary and Transition Table of the Impact of Event Scale-Revised (IES-R) During the Study Time Frame

eTable 2. Pearson's Correlation Coefficient Matrix Within the Subscale of the General Health Questionnaire (GHQ)

eTable 3. Comparison of Variables Between Participants Who Provided Data Once and Those Who Provided Data Twice or More

eFigure 1. Time Transformation in the Dataset With Deployment and Follow-Up-Collected Time-to-Event Times

eFigure 2. Flowchart of the Selection of Participants for the Analysis

eFigure 3. Fit Indices of the Impact of Event Scale-Revised (IES-R) Assessments in Latent Growth Mixture Models (LGMM)

This supplementary material has been provided by the authors to give readers additional information about their work.

eTable 1. Summary and transition table of the Impact of Event Scale Revised (IES-R) during the study time frame

Month	6	12	18	24	30	36	42	48	54	60	66	72	78
n	1,173	1,218	1,157	1,093	930	935	884	686	634	344	373	227	164
Median (IQR)	0.00 (0.00, 3.00)	0.00 (0.00, 2.00)	0.00 (0.00, 2.00)	0.00 (0.00, 2.00)	0.00 (0.00, 2.00)	0.00 (0.00, 1.00)	0.00 (0.00, 3.00)	0.00 (0.00, 1.00)	0.00 (0.00, 2.00)	0.00 (0.00, 1.00)	0.00 (0.00, 1.00)	0.00 (0.00, 1.00)	0.00 (0.00, 2.00)
Mean	2.94	2.39	2.77	2.45	2.71	1.95	2.81	1.79	2.58	1.70	1.28	1.60	2.17
Responded 2 times (n (%))	484 (41.3)	360 (29.6)	288 (24.9)	331 (30.3)	216 (23.2)	112 (12.0)	59 (6.7)	52 (7.6)	27 (4.3)	13 (3.8)	4 (1.1)	4 (1.8)	4 (2.4)
Responded 3 times (n (%))	260 (22.2)	343 (28.2)	297 (25.7)	300 (27.4)	167 (18.0)	316 (33.8)	222 (25.1)	162 (23.6)	84 (13.2)	55 (16.0)	33 (8.8)	18 (7.9)	14 (8.5)
Responded 4 times (n (%))	205 (17.5)	320 (26.3)	239 (20.7)	269 (24.6)	261 (28.1)	309 (33.0)	278 (31.4)	274 (39.9)	217 (34.2)	128 (37.2)	93 (24.9)	68 (30.0)	35 (21.3)
Responded 5 times (n (%))	187 (15.9)	151 (12.4)	250 (21.6)	149 (13.6)	203 (21.8)	154 (16.5)	242 (27.4)	154 (22.4)	223 (35.2)	104 (30.2)	160 (42.9)	93 (41.0)	65 (39.6)
Responded 6 times (n (%))	37 (3.2)	44 (3.6)	83 (7.2)	44 (4.0)	83 (8.9)	44 (4.7)	83 (9.4)	44 (6.4)	83 (13.1)	44 (12.8)	83 (22.3)	44 (19.4)	46 (28.0)

The upper table presents the median (IQR) and mean of the IES-R at each time point. The lower table presents the number of responses (%) at each time point. For instance, there were 977 participants who responded 2 times, 757 participants who responded 3 times, 674 participants who responded 4 times, 427 participants who responded 5 times, and 127 participants who responded 6 times. The total number of responses for the '2 times' row is 1,954, which equals twice the number of participants who responded 2 times. For example, the cell at the intersection of the '2 times response' row and '6 months' column shows 484 responses from participants who responded 2 times. The rate of 2 times responses is then calculated as 41.3% of the total responses at the 6-month mark.

IES-R, Impact of Event Scale-Revised; %, response rate at each time point; IQR, interquartile range (25%, 75%)

© 2024 Kitano et al. *JAMA Network Open.*

eTable 2. Pearson's correlation coefficient matrix within the subscale of the General Health Questionnaire (GHQ)

	Somatic symptom	Sleep disturbance	Social dysfunction	Anxiety and dysphoria	Suicidal depression
**General illness**	.57	.54	.40	.52	.33
**Somatic symptom**		.51	.23	.47	.37
**Sleep disturbance**			.31	.53	.36
**Social dysfunction**				.33	.23
**Anxiety and dysphoria**					.49

Pearson's correlation coefficients were calculated between all subscales of the GHQ. n = 2,962. All coefficient showed P < 0.001.

GHQ, General Health Questionnaire

eTable 3. Comparison of variables between participants who provided data once and those who provided data twice or more

		Once (n = 504)	Twice or more (n = 2,962)	p-value
**Age (mean (SD))**		32.6 (8.9)	33.9 (7.2)	< .001
**Sex (n (%))**	**Male**	480 (95.2)	2,901 (97.9)	< .001
	**Female**	24 (4.8)	61 (2.1)	
**Rank (n (%))**	**Sergeant**	294 (58.3)	2,205 (74.4)	< .001
	**Officer**	126 (25.0)	536 (18.1)	
	**Private**	84 (16.7)	221 (7.5)	
**General illness (mean (SD))**		8.0 (2.1)	8.2 (2.0)	.12
**Somatic symptom (mean (SD))**		6.5 (1.9)	6.5 (1.9)	.96
**Sleep disturbance (mean (SD))**		7.5 (2.4)	7.7 (2.5)	.11
**Social dysfunction (mean (SD))**		9.4 (1.7)	9.4 (1.6)	.34
**Anxiety and dysphoria (mean (SD))**		7.2 (2.3)	7.2 (2.3)	.93
**Suicidal depression (mean (SD))**		5.5 (1.3)	5.5 (1.3)	.99
**IES-R (mean (SD))**		2.6 (5.9)	2.8 (6.2)	.58
**p-PTSD (n (%))**	**Yes**	10 (2.0)	53 (1.8)	.90
	**No**	494 (98.0)	2,909 (98.2)	

Each continuous variable was compared between those with data from only one time point (excluded in this study) and those with data from two or more time points (included in this study) by conducting a t-test. In addition, the frequency of each categorical variable was compared between the two groups by conducting a chi-square test. We chose the first IES-R data for each participant for these analyses in the "Two times or more" group. Consequently, the number of current p-PTSD cases was 53, compared to a cumulative total of 117 p-PTSD cases.

IES-R, Impact of Event Scale-Revised; p-PTSD, probable post-traumatic stress disorder.

© 2024 Kitano et al. *JAMA Network Open.*

eFigure 1. Time transformation in the dataset with deployment and follow-up-collected time-to-event times

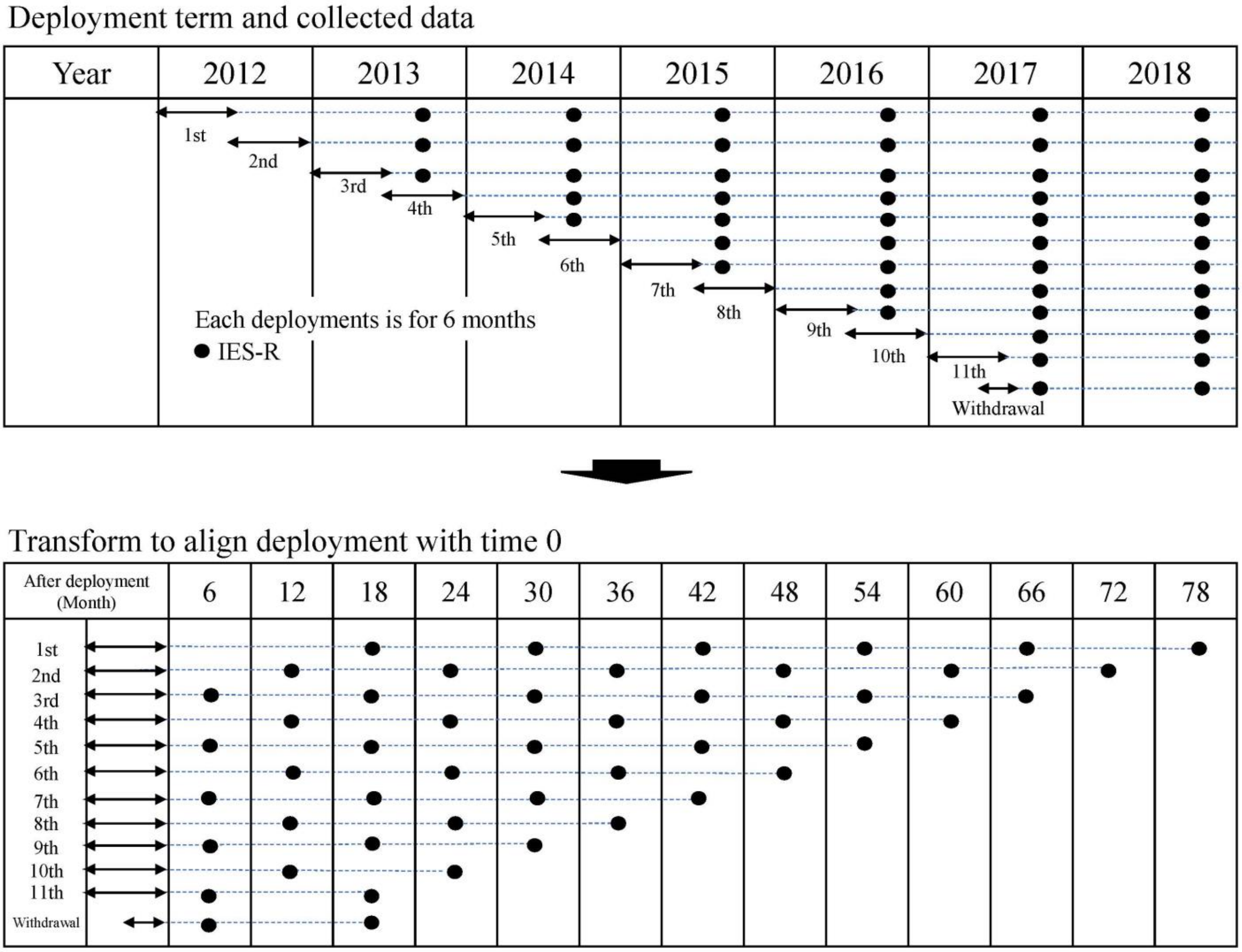

These charts display unit deployment. The upper chart shows the deployment term for each unit with the data collection points marked by black dots. The lower chart shows the same information but aligns the mission completion of the deployment to time zero. Months represent the elapsed time after returning from deployment.

© 2024 Kitano et al. *JAMA Network Open.*

eFigure 2. Flowchart of the selection of participants for the analysis

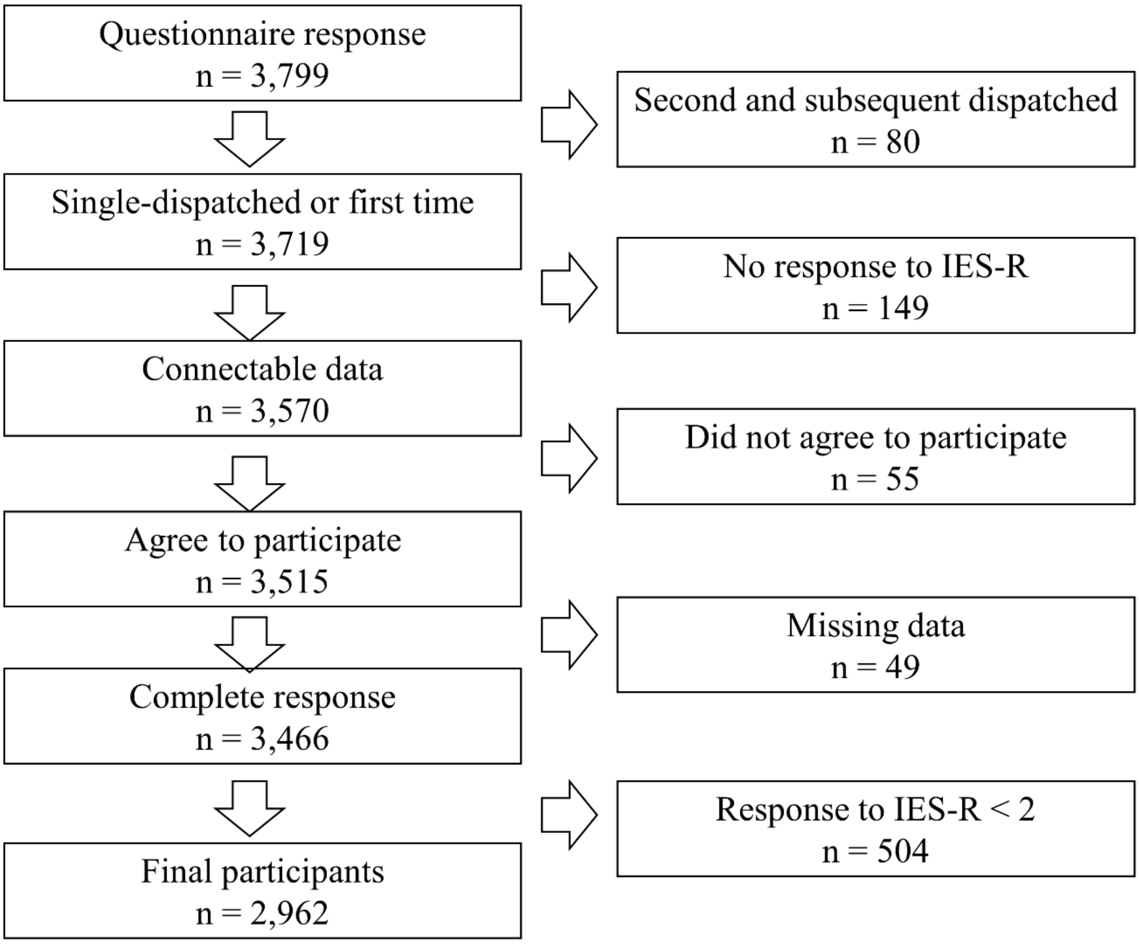

© 2024 Kitano et al. *JAMA Network Open.*

eFigure 3. Fit indices of the Impact of Event Scale-Revised (IES-R) assessments in latent growth mixture models (LGMM)

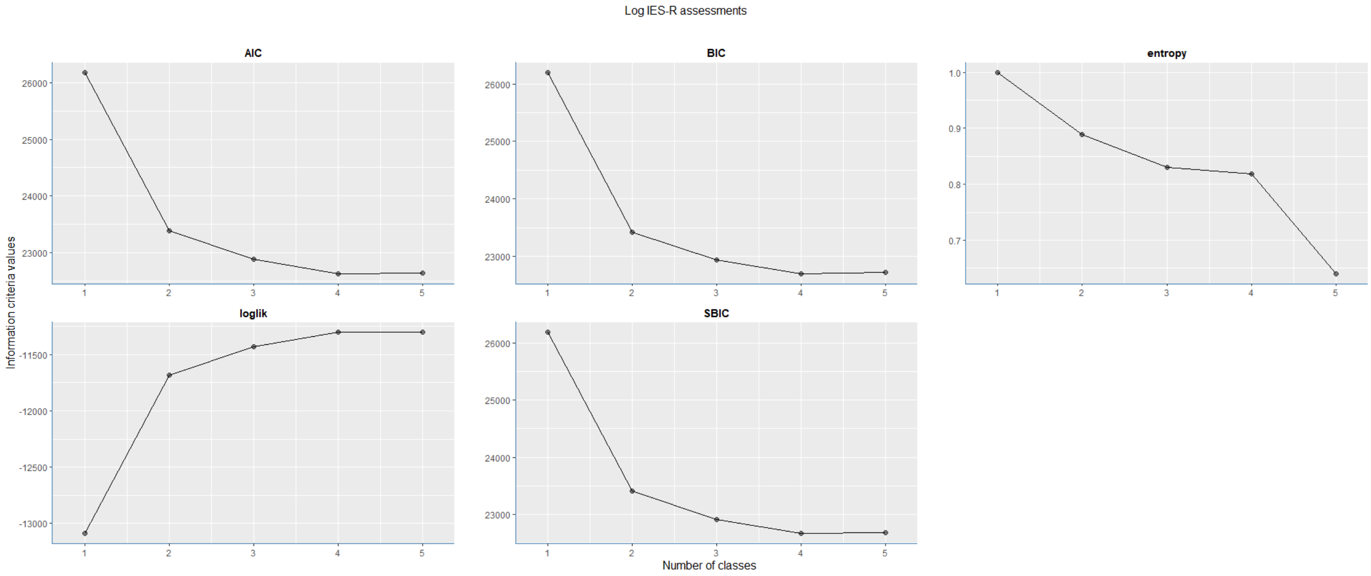

	G	loglik	conv	npm	AIC	BIC	SBIC	entropy	%class1	%class2	%class3	%class4	%class5
hlme1	1	-13090.4208524114	1	3	26186.8417048228	26204.8225648072	26195.290428291	1	100	NA	NA	NA	NA
hlme2	2	-11685.3573234642	1	6	23382.7146469284	23418.6763668974	23399.6120938648	0.888496621734315	84.3686698176907	15.6313301823093	NA	NA	NA
hlme3	3	-11432.6466925253	1	9	22883.2933850506	22937.2359650041	22908.6395554552	0.830492517130455	74.2403781228899	6.2457798784605	19.5138419986496	NA	NA
hlme4	4	-11300.1730871587	1	12	22624.3461743174	22696.2696142553	22658.1410681902	0.818709463930558	72.3497636731938	16.1715057393653	6.14449696151249	5.33423362592843	NA
hlme5	5	-11300.1730871587	1	15	22630.3461743174	22720.2504742398	22672.5897916584	0.640199728068895	6.14449696151249	71.4382174206617	5.50303848750844	0	16.9142471303174

The best fit was determined by the lowest AIC, BIC, and SBIC values, where the entropy values approached one.

AIC, Akaike information criterion; BIC, Bayesian information criterion; Loglik, Log-likelihood; SBIC, Sample-size-adjusted BIC.

© 2024 Kitano et al. *JAMA Network Open.*
