# Supplementary material for: Symptoms of Posttraumatic Stress Disorder Among Japanese Peacekeepers Deployed in South Sudan
Source: JAMA Netw Open. 2024 Jul 24;7(7):e2424388. doi: 10.1001/jamanetworkopen.2024.24388 (PMC11270132; doi:10.1001/jamanetworkopen.2024.24388)
Supplement: Supplement 2. — Data Sharing Statement [file jamanetwopen-e2424388-s002.pdf]

Data Sharing Statement

Kitano. Symptoms of Posttraumatic Stress Disorder Among Japanese Peacekeepers Deployed in South Sudan. *JAMA Netw Open*. Published July 24, 2024.
doi:10.1001/jamanetworkopen.2024.24388

Data
Data available: No

Additional Information
Explanation for why data not available: Since the data was collected based on the Japan Self-Defense Forces' policy, we basically do not disclose it, but we can consider data sharing depending on the need and use of the data.
